# Supplementary figures and images for: The association of macronutrients in human milk with the growth of preterm infants
Source: PLoS One. 2020 Mar 26;15(3):e0230800. doi: 10.1371/journal.pone.0230800 (PMC7098608; doi:10.1371/journal.pone.0230800)

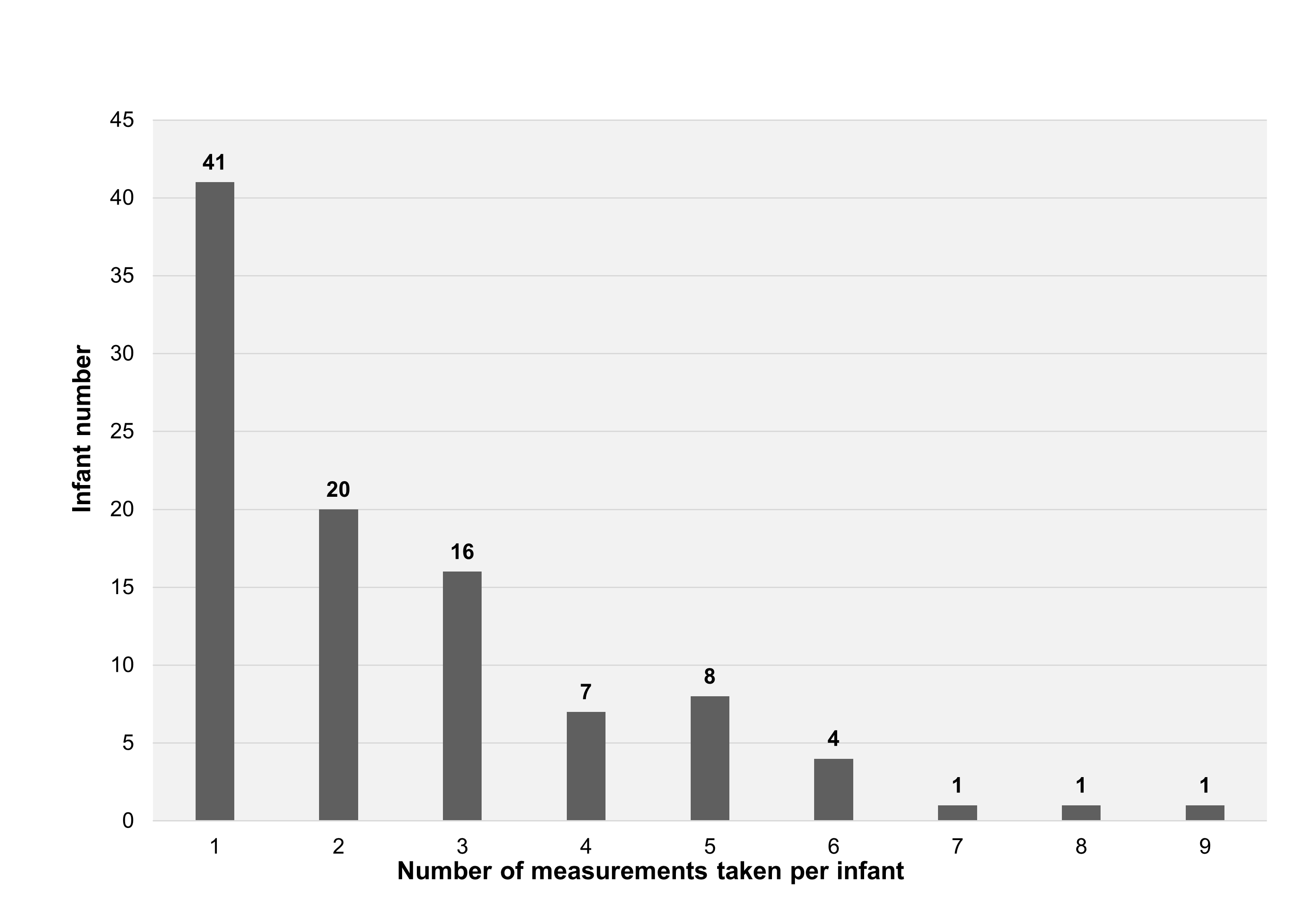

Supplement: S1 Fig — (TIF) [file pone.0230800.s002.tif]
