# Supplementary material for: The association of macronutrients in human milk with the growth of preterm infants
Source: PLoS One. 2020 Mar 26;15(3):e0230800. doi: 10.1371/journal.pone.0230800 (PMC7098608; doi:10.1371/journal.pone.0230800)
Supplement: S1 Table — (DOCX) [file pone.0230800.s003.docx]

**S3 Table.** **Demographic data for included infants**

|  | Infants (N=99) | | |
| --- | --- | --- | --- |
| Variables | mean | ± | SD |
| Gender |  |  |  |
| Male | 49 (49.5%) | | |
| Female | 50 (50.5%) | | |
| Multiple Birth |  | | |
| Singleton | 67(67.7%) | | |
| Twins | 28(28.3%) | | |
| Triplets | 4(4.0%) | | |
| Small for gestational age | 16 (16.2%) | | |
| IVH (≥ grade 2) | 4 (4.0%) | | |
| Significant PDA | 6 (6.1%) | | |
| Necrotizing enterocolitis | 18 (18.2%) | | |
| Gestational age at birth, weeks | 30.4 | ± | 2.9 |
| Extremely preterm (<28 weeks) | 25 (25.3%) | | |
| Very preterm (28 to 32 weeks) | 34 (34.3%) | | |
| Moderate to late preterm (32 to 37 weeks) | 40 (40.4%) | | |
| Birth body weight, g | 1364.7 | ± | 411.5 |
| Birth Body weight, Z-score | -0.5 | ± | 1.0 |
| Duration of TPN support, day | 14 (31) ^&^ | | |

IVH: intraventricular hemorrhage; PDA: patent ductus arteriosus; TPN: total parenteral nutrition; SD: standard deviation

^&^ The variable is reported as the median (interquartile range).
