# Supplementary material for: The association of macronutrients in human milk with the growth of preterm infants
Source: PLoS One. 2020 Mar 26;15(3):e0230800. doi: 10.1371/journal.pone.0230800 (PMC7098608; doi:10.1371/journal.pone.0230800)
Supplement: Attachment — Submitted filename: Response to Reviewers.docx [file pone.0230800.s004.docx]

Dear Dr. Young:

Thank you for inviting us to submit a revised draft of our manuscript entitled, “The impact of macronutrients in human milk on the growth of preterm infants” to PLUS ONE. We also appreciate the time and effort you and each of the reviewers have dedicated to providing insightful feedback on ways to strengthen our paper. Thus, it is with great pleasure that we resubmit our article for further consideration. We have incorporated changes that reflect the detailed suggestions you have graciously provided. We also hope that our edits and the responses we provide below satisfactorily address all the issues and concerns you and the reviewers have noted.

To facilitate your review of our revisions, the following is a point-by-point response to the questions and comments.

**To Reviewer #1:**

**Thank you for your comments and suggestions. The questions are answered below:**

1. **RE: The only components analyzed are protein, fat, and carbohydrates in human milk with a commercial analyzer, apparently without quality controls. The composition of human milk was in-fact not analyzed in this study as claimed by the authors.**

**Ans:** We agree with you. There are many important compositions of human milk, and we only could analyze the macronutrient levels. I’m sorry to mislead about that. I have removed the word “composition” and replaced it with “macronutrients” in some paragraph (Line 33, 77,240). MIRIS human milk analyzer was proved a reliable and helpful tool for bedside analysis of human milk [18-21]. We also added explanation about quality control in the methods section as follows: “The check procedure with zero level adjustment and the replicate samples control was required quality controls of the MIRIS HMA. A zero-setting check procedure was implemented routinely at start-up and after cleaning the instrument, i.e. every 10th analysis. A monthly control of repeatability test was also performed.” (Page 9, Line 117-121)

2. **RE: It is not clearly described what milk was analyzed other than human milk. What type of donor milk was used? What is the difference between human and maternal milk as all the milk is human milk?**

**Ans:** I’m sorry about the unclear description. All milk analyzed in this study was human milk. There were two source of human milk, milk of infants’ own mothers and pasteurized donor milk. We added some explanation about the donor milk as follows: “All donor human milk fed to infants in this study was obtained from the Taipei City Hospital Milk Bank (TCHMB) which was established in 2005. The qualified donor milk was pasteurized (62.5°C for 30 minutes). Milk donors were unpaid volunteers, most of whom delivered term infants (91.2%). Most donor milk is mature milk because up to 96.5% mother donated their human milk while postpartum >1 month (96.5%).[18]” (Page 7-8, Line 93-98)

3. **RE: Infants were included only when exclusively fed with human milk of sorts without parental nutrition support, which has not been defined and it is up for the reader to guess. Yet, all over the manuscript it is indicated that there was parental nutrition support.**

**Ans:** I had reworded the sentence in the abstract to clarify that infants who received parental nutrition previously were eligible; the inclusion criterion was not be receiving parental nutrition at the time the sample was taken. (Page 3, Line 36-39)

4. **RE: The sample collection details are missing, and no ex- or inclusion criteria are provided for the mothers.**

**Ans:** We added a new figure (Figure 1) to illustrate the inclusion /exclusion criteria and the participants flow through the trial. (Section 2. Materials and Methods, Page 7, Line 82-91). The sample collection details were also added in Figure 2. Time relationship between milk analysis and growth measurement.

5. **RE: Statistical analysis was adjusted for numerous factors which are not explained to why they were chosen nor how they were choses or tested to be included. Yet, infant age was not one of them.**

**Ans:** We redid the statistical analysis and included gestational age and post menstrual age (PMA) as cofactors (section 2.4 cofactors, Page 11, Line 155-159). The results were showed in Table 4 and Table 5.

6. **RE: We don’t know whether the data was normally distributed, while the used tests in the paper are based on normally distributed variables.**

| Kolmogorov–Smirnov test | | |
| --- | --- | --- |
| Variables | D | P value (Pr>D) |
| Protein concentration (g/100ml) | 0.052 | 0.098 |
| Total protein intake amount (g/kg/day) | 0.050 | 0.134 |
| Body weight gain velocity (g/kg/day) | 0.045 | >0.150 |

**Ans:** We added the description about normally distribution in the section 2.5 Statistical analysis as follows: “In order to test whether the data are normally distributed, the Kolmogorov–Smirnov test was used. The variables included protein concentration of human milk, total protein intake amount, and body weight gain velocity were all normally distributed.” (Page 11, Line 164-167) The Kolmogorov–Smirnov test results are displayed as follows:

7. **RE: The reader is not informed about how long milk samples were collected (lactation stage/infant age).**

**Ans:** We added new **figure 2** to illustrate about time relationship between milk analysis and growth measurement (Page 8, Line 106-110). The gestational age at birth and infant age (PMA) at the time of milk samples collection were reported in **table 1** (page 13-14, Line 186). Because we only enrolled the preterm infants who reached full enteral feeding, most human milk samples analyzed in the study were mature milk.

8. **RE: The authors emphasize the daily protein intake as being correlated to infant weight gain, which is only a derivative of the protein in the milk, which was found to correlate in the first place. None of the found results are novel or unexpected.**

**Ans:** We agree with your assessment. The crucial importance of the protein amount for the growth of in-hospital premature babies has been established. However, most studies were conducted in the late 20^th^ century in Western countries. The clinical practice and nutrition guideline have changed much over time. Otherwise, most of these previous studies focused on the impact of parenteral nutrition in the early growth stage of premature infants, and they assumed the human milk energy content of 67 kcal/100 ml and protein content of 1.2-1.4g/100ml. The strength of our study is that we determined the actual level of human milk macronutrients based on daily milk analyses, and the detailed feeding amount including fortifier dosage was recorded to accurately calculate the total daily protein and energy intake. In addition, we only enrolled preterm infants who had reached full enteral feeding with human milk to eliminate the effect of parenteral nutrition or infant formula. We conducted a domestic and modern study in an East Asian population. (Page 23-24, Line 308-316)

9. **RE: The figure has no title and no explanation is given, e.g. what are the 3 lines in each of the graphs?**

**Ans:** The figure was relabeled as figure 3. The title of figure was displayed in the manuscript, and we added figure legend below it (Figure 3, Page 17, Line 207-211).

10. **RE: Table 3 is not useful for the reader; the data of the significant correlation is much better displayed in a graph.**

**Ans:** The significant correlation of protein concentration of HM and weight gain was displayed in figure 1. There was no significant correlation between other human milk macronutrients and the growth, so these data were not shown in the graph.

11. **RE: The objective of your research is not a strength of this study.**

**Ans:** Thank you for your important suggestion. We reworded the first sentence of objective to clarify that we only analyzed the macronutrients of human milk and the correlations with the growth of in-hospital preterm infants. (Page 3, Line 33)

12. **RE: These are just some of the concerns I have for this manuscript, besides the needed language revisions and corrections, e.g. p7L100: you are not sampling human milk fed infants, you are collecting milk that is fed to infants.
    Ans:** Thank you for your comments. I had revised this sentence as your suggestion (Page 8, Line 103-104). Language revisions and corrections of all over manuscript were also done (section Acknowledgement, line 324-325)

**To Reviewer #2:**

**Thank you for your comments and suggestions. The questions are answered below:**

1. **RE: Should give age and weight status (z-score) of infants at time of growth measurements to delineate the degree of EUGR**

**Ans:** Infant age (post menstrual age) and weight status (z-score) at time of growth measurements were added in Table 1. We also used the change in weight z-score within 1 week to evaluate the weight gain velocity as follows (figure c and d). But the correlation between weight z core change and protein intake is weak, probably because the difference in z score was subtle within a short period (one week). So the results was not shown in figure 1.

2. **RE: Line 106-107: Must state length of period for which weight gain is given. "Weekly average" is too vague.**

**Ans:** We have included a new Figure 2 (Page 8, Line 110) to further illustrate the time relationship between milk analysis and growth measurement.

3. **RE: Need to give time relationship between milk analysis and growth measurement.**

**Ans:** We have added new Figure 2 (Page 8, Line 110) to further illustrate it. Please see RE#2 above.

4. **RE: Need to point out that fortified and unfortified milk was analyzed**

**Ans:** All analyzed human milk is unfortified milk. Before fed to the infants, Human milk fortifier was added as tolerated with a maximum of 4 packets per 100ml in the bedside. The brand and dosage of human milk fortifier (Similac HMF or Enfamil HMF) were also recorded, and then total protein amount (g/kg/d) and protein-energy ratio (g/100kcal) were calculated. We have supplemented the section 2.2 Enteral Feeding data with explanations of human milk fortifier (Page 9-10, Line 130-133)

5. **RE: Lines 100-103: Milk analysis: of two measurements in a given day, the average was used. But then how were "weekly averages" calculated?**

**Ans:** We have added new Figure 2 (Page 8, Line 110) to further illustrate it. Please see RE#2 above. In consideration of the wide variation of human milk composition, ie. more lipid content in hindmilk, we collected the human milk that was fed to infants twice a day (Page 8, Line 102-106). Then we used the weekly average data to reduce the effect of day-to-day variance of body weight measurements. (Page 10, Line 139-141).

6. **RE: Lines 145-146: Need not repeat in text what the table says**

**Ans:** Thank you for your suggestion. We have deleted these sentences about Table 2.

7. **RE: Line 231: What is "HMA"?**

**Ans:** I’m sorry about the unclear description. I had corrected the word with “MIRIS HMA” in the manucript. MIRIS HMA is an abbreviation of MIRIS human milk analyzer, which explained in page 9 line 115.

8. **RE: Discussion is too long. Quote only articles that are directly related to the manuscript**

**Ans:** Thank you for your comment. We have removed some references and compressed the discussion.

9. **RE: "Breastfeeding, Breast Feeding and Maternal Nutrition" should be deleted from key words**

**Ans:** We have removed these key words.

**To Reviewer #3**

**Thank you for your comments and suggestions. We have incorporated these suggestions throughout our paper. The details are answered below:**

**MAJOR SUGGESTIONS:**

1. **RE: Please report the total number of infants included in the study, and the number of measurements taken per infant.**

**Ans:** Total 99 infants was included in the study (Page 7, Line 88-89). Each infant may contribute multiple data points if long hospitalization period. Total 245 weekly data were collected (Page 8, Line 105-106). The number of measurements taken per infant was displayed as follow:

| Number of measurements taken per infant | Infant number |
| --- | --- |
| 1 | 41 |
| 2 | 20 |
| 3 | 16 |
| 4 | 7 |
| 5 | 8 |
| 6 | 4 |
| 7 | 1 |
| 8 | 1 |
| 9 | 1 |

We also added new Figure 1 and Figure 2 to illustrate the participants flow and collection sample details.

2. **From the authors' description it seems that each baby could contribute multiple data points (e.g. if a baby was in the hospital for 5 weeks it could contribute 5 different data points to the study, an average milk content and average growth rate for every week). If multiple data points from the same baby were used in the analysis, then the data aren't independent and the statistical analysis needs to account for that (for example, using generalized estimating equations to account for** **correlated data due to repeated measurements on the same infant). Alternatively, the authors could consider using the average protein intake and growth rate for each baby over the entire study period so that each infant contributes just one data point and adjusting for length of stay as a covariate.**

**Ans:** You have raised an important question. In fact, some infants contributed multiple data points in this study as the description RE#1 above. We used generalized estimating equations to account for these correlated data. The results were displayed in Table 4 and table 5. We also supplemented the 2.5 statistical analysis section with explanations of generalized estimating equations (Page 12, lines 169-171).

3. **RE: The authors should investigate adding gestational age at birth as a covariate. Gestational age is associated with protein content in maternal milk and with growth, and could act as either a confounder, a mediator, or possibly an effect modifier.**

**Ans:** We agree with you. Gestational age at birth is an important covariate. We redid the statistical analysis and included gestational age and post menstrual age (PMA) as cofactors (section 2.4 cofactors, Page 11, Line 155-159). The results were displayed in Table 4 and Table 5.

**MINOR SUGGESTIONS:**

**OVERALL:**

1. **RE: The authors refer to "catch-up growth" and "catch-up growth stage" throughout the paper. Can they clarify what they mean by this (crossing percentiles upward on the growth chart? rapid growth? a critical period for growth?) and perhaps use a more specific term?**

**Ans:** We agree with you. There is no unified definition of catch-up growth. Catch-up growth is usually defined as reaching an SD score of 1 –2 SDS of the reference population, but in some studies a change >0.67 SD has been used as cut-off. In our paper, I thought catch-up growth stage is a rapid growth period after initial postnatal growth failure. We have tried to avoid this unspecific term in our paper.

**ABSTRACT:**

1. **RE: In the methods section, I suggest rewording the first sentence to clarify that infants who received PN previously were eligible; the criterion was to not be receiving PN at the time the sample was taken.**

**Ans:** Thank you for your suggestion. I have modified the sentence (Page 3, Line 36-39)

2. **RE: I also recommend reporting the number of infants in the study in addition to the total number of milk samples, and adding the effect size of the association between protein intake and growth in addition to the p-value.**

**Ans:** Total 99 infants was enrolled in the study, which was displayed in abstract (Line 35), section 2 Materials and Methods (Line 89) and figure 1. We set a power of 90% (description in section 2.5 Statistical analysis, line 172), and the effect sizes were 5.70 and 2.28 (reported in section 3 Results, Line 199 and 202).

3. **RE: In the conclusion, the statement “protein intakes have positive impact on the body weight gain” sounds causative but this study only shows associations; I suggest rewording this statement.**

**Ans:** We have revised the conclusion as follows: “Both the protein concentration of human milk and the daily total protein intakes have positive correlations with the body weight gain of prematurity.” (Page 4, Line 51-53)

**INTRODUCTION: This is a clear and concise summary of the relevant background literature and nicely describes the research question.**

1. **RE: I would suggest adding a hypothesis.**

**Ans:** We have added our hypothesis and revised the last part of introduction (Line 75-78).

2. **RE: There are a few minor grammatical points: Line 71, change “prognosis” to “prognostic”. Line 72, add “of” between “especially” and “poor neurodevelopmental outcomes.”**

**Ans:** Thank you for your suggestion. I have corrected these grammatical errors.

**METHODS:**

1. **RE: Can the authors comment on what proportion of eligible infants were included in the study, and if any were excluded what the reasons for exclusion were?**

**Ans:** We added a new figure (**Figure 1**) to illustrate the inclusion /exclusion criteria and the participants flow through the trial. The description was also added in Section 2. Materials and Methods (Page 7, Line 72-89).

2. **RE: The authors should describe how they chose the two-point approach to measuring growth velocity from among the alternative methods available (eg Patel et al, Calculating Postnatal Growth Velocity in Very Low Birth Weight (VLBW) Premature Infants, J Perinatal 2009).**

**Ans:** This is an important question. There was no standardization of methods used to calculate preterm infant growth velocity. According to a systematic review (Fenton TR, et al. Preterm Infant Growth Velocity Calculations: A Systematic Review. Pediatrics. 2017), the most frequently used method reported in the 1543 studies was g/kg/d (40%), followed by g/d (32%); 29% reported change in z score. The majority of studies that reported g/kg/d calculations did not report what they used for the weight as denominator (n = 94 [62%]). Of the studies that reported the denominators, the majority used an average of the start and end weights as the denominator (36%) followed by exponential methods (23%), and birth weight (10%). So we chose the most common method (two-point approach) and the z-score change to calculate growth velocity. Thank you for the literaure you provided (Patel et al, Calculating Postnatal Growth Velocity in Very Low Birth Weight (VLBW) Premature Infants, J Perinatal 2009). It seems that exponential method is superior (more accurate). We will consider use this method to evaluate the GV in the future. We have rewritten the section 2.4 outcome measurement for further explaination and cited these references.

Otherwise, we also used the change in weight z-score within 1 week to evaluate the weight gain velocity as follows (figure c and d). But the correlation between weight z core change and protein intake is weak, probably because the difference in z score was subtle within a short period (one week). So the results was not shown in figure 1.

3. **RE: Please describe how extreme values were handled. In my experience, there are often a few extreme values in any relatively large dataset of milk samples analyzed by MIRIS (often due to the sample not being fully homogenized). Were there any extreme values and if so were they included or discarded? If discarded, what criteria were used to determine which samples were excluded?**

**Ans:** We agree with your experience. These extreme values were included and analyzed in our study. We hoped we could reduce the effect of extreme values by collecting more samples and calculating the weekly average (every weekly data consists of 14 milk samples).

4. **RE: Describe what is meant by the covariate "bottle feeding" - is that bottle feeding on the day the sample was taken? Bottle feeding ever? Similarly, describe what is meant by "ventilator support" - on the day of the sample? Ever?**

**Ans:** We observed that bottle feeding may consume more energy than feeding via a nasogastric tube and may affect infant’s growth in the clinical practice. Otherwise, ventilator support may reduce the breathing work and the energy consumption of infants. So, we added these 2 cofactors in the model. Both “bottle feeding” and “ventilator support” meant that was used in some days of the week when the sample was taken. We added the above explanation below table 4 and table 5 (Line 224-226, 233-235)

5. **RE: The authors state in the discussion that they could not use length or head circumference (HC) measurements due to measurement error. It may be worth adding to the methods section that the intention was to assess length and HC, and how they determined that the measurements were too inaccurate to use.**

**Ans:** We have added this to method section (Line 143-145) and explanation about inaccuracy in discussion section (Line 293-299) as follows” Firstly, we measured the head circumference (HC) and body length weekly, and calculated growth velocity as centimeters/week (cm/wk). However, some of the growth velocity (cm/wk) was minus. The possible cause is that the time interval is too short to show the subtle change in HC and length. Due to inaccurate measurement, we didn’t analyze the relationship between HC, length growth and daily protein intake.”

**RESULTS:**

1. **RE: In Table 1: The title of the right column is "weekly sample" - so are these results describing individual infants, or total samples (eg were 60% of infants in the study male or 60% of samples came from male infants?). Also clarify what is meant by "Post menstrual age;" is this the PMA at the time the sample was taken? For duration of TPN support, I suspect** **the variable is quite skewed; consider reporting median rather than mean and standard deviation.**

**Ans:** I’m sorry about the unclear description. The results of table 1 are description total weekly sample (n=245). For example, 61.2% of weekly data came from male infants. We also added explanation (*) for PMA and z-score below table 1 as follow: PMA and z-score represented the infant age and weight status at time of growth measurements. According to your suggestion, we have reported the variable of TPN duration as median value in table 1 and added the description in section 2.5. statistical analysis (Page 11, Line 162-164)

2. **RE: Can the authors comment on the proportion of samples that were donor milk versus maternal milk? There are well described differences between the macronutrient content of donor vs maternal milk, as the authors have described in the discussion.**

**Ans:** The proportion of samples that were donor milk (12.7%) was added in Table 1. It means total or part of milk samples were donor milk within 1 week.

3. **RE: Line 52, remove "Nevertheless"**

**Ans:** Thank you for your suggestion. We have removed it.

**DISCUSSION: Overall the organization of the discussion could be improved, and the authors could more clearly state how their findings add to what is already known on this topic. Also, while the remainder of the paper has excellent English language, the discussion has a few minor English errors that should be corrected; I have attempted to note them here to assist the authors in clarifying their language.**

1. **RE: The first paragraph is very long. Consider breaking it up into several paragraphs and clearly identify the key point of each paragraph.**

**Ans:** We have broken it up to three paragraph and compressed the section of discussion.

2. **RE: Line 187, add that the linear association was positive. Line 190 clarify what "it" refers to. Line 191, consider changing "amount" to "content of milk" or "intake" depending on the authors' intention**

**Ans:** Thank you for your comment. We have reworded that as above. (Page 20, Line 238, 241-242)

3. **RE: Lines 211-212: clarify what "higher" means (higher than what?)**

**Ans:** Thank you for your suggestion. We had compressed the section of discussion and deleted this paragraph.

4. **RE: Line 222, consider describing the protein content of the HMF rather than the number of packs**

**Ans:** We have added the protein content of the HMF (1.0-1.1g/100ml) in Line 274.

5. **RE: Line 227, clarify if the authors are referring to the current study or to previous studies. Lines 227-235; this paragraph would benefit from better organization to determine the authors' key point**

**Ans:** We removed this paragraph due to little correlation with our study.

6. **RE: Line 237, I think the authors mean "protein intake" rather than "fortification" here**

**Ans:** We have rewritten this sentence (Line 277-278).

7. **RE: Line 257, by "was not provided in our results" do the authors mean it was not found in their analysis or was not studied?**

**Ans:** We meant that it was not studied (analyzed). We have deleted this sentence due to vague description.

8. **RE: Correct the English language: Line 189, remove "the" before growth. Line 198, "observation" should be "observational." Lines 201-202 I would suggest "Clinical practice and nutrition guidelines have changed substantially over the past two decades." Line 207 change "the human milk sample" to "human milk samples." Lines 211-212, consider changing "approximate" to "support," and add "the" before "intrauterine fetus." Line 217, add "with" before "body weight." Line 221, change "while" to "when." Line 240, "systemic" should be "systematic." Line 251, "target" should be "targeted." Line 254, "consist" should be "exist." Line 260, "group" should be "groups." Lines 263-264 need to be rewritten and clarified. Line 277, "prematurity" should be "premature infants"**

**Ans:** Thank you for these comments. We have corrected these language errors. The whole manuscript was edited for language and writing (section Acknowledgement, Line 319-320)

9. **Can the authors comment on generalizability of their results based on the inclusion criteria of their study?**

**Ans:** We have added the description about generalizability as follows: “Finally, we only enrolled in-hospital premature infants with a gestational age of 25 to 36 weeks who reached full enteral feeding and under exclusive human milk diet. Whether the protein content in human milk also plays a crucial role in the premature infants under mixed feeding with formula milk, with parental nutrition support, and the infants after hospital discharge, remains to be determined.” (Line 302-307)

Again, thank you for giving us the opportunity to strengthen our manuscript with your valuable comments and queries. We have worked hard to incorporate your feedback and hope that these revisions persuade you to accept our submission.

Sincerely,

Yi-Hsuan Lin, MD

Corresponding Author

Ming-Chih Lin, M.D. Ph.D.

Division of Neonatology & Pediatric Cardiology

Department of Pediatrics,

Taichung Veterans General Hospital,

Taichung city, Taiwan

TEL: 886-4-23592525-5901

FAX: 886-4-23741359
