# Supplementary material for: The association of macronutrients in human milk with the growth of preterm infants
Source: PLoS One. 2020 Mar 26;15(3):e0230800. doi: 10.1371/journal.pone.0230800 (PMC7098608; doi:10.1371/journal.pone.0230800)
Supplement: Attachment — Submitted filename: Response to Reviewers.docx [file pone.0230800.s005.docx]

Dear Dr. Young:

Thank you for inviting us to submit a revised draft of our manuscript entitled, “Association of macronutrients in human milk with the growth of preterm infants” to PLOS ONE again. We also appreciate the time and effort you and each of the reviewers have dedicated to providing insightful feedback on ways to strengthen our paper. Thus, it is with great pleasure that we resubmit our article for further consideration. We have incorporated changes that reflect the detailed suggestions you have graciously provided. We also hope that our edits and the responses we provide below satisfactorily address all the issues and concerns you and the reviewers have noted.

To facilitate your review of our revisions, the following is a point-by-point response to the questions and comments.

**To Reviewer #3:**

**Thank you for your comments and suggestions. The questions are answered below:**

MAJOR ISSUES:
STATISTICAL ANALYSIS

1. **RE: All analyses need to account for correlated data. The analysis of protein intake and weight gain (Table 5) still states it is linear regression rather than generalized estimating equations.**

Ans: I’m sorry about the wrong title of table 5. The result of table 5 is from a generalized estimating equation. I have corrected the title of table 5 (Page 19, Line 243)

2. **RE: The authors report measuring changes in weight Z-score (p 10, line 138) but I do not see any reported results using weight Z-scores. Either this line should be removed, or the authors should report the Z-score results.**

Ans: The correlation between weight z core change and protein intake is weak, probably because the difference in z score was subtle within a short period (one week). So the results was not reported. I have removed the description in section method (p 10, line 141-142).

3. **RE: The authors should describe why they chose the covariates included in the models (eg a prior based on known associations with infant growth and milk macronutrient content, or what criteria they used if the covariates were chose in some other fashion).**

Ans: We added the description as follows, “Univariate analysis for possible confounding factors was performed. All the factors significant (p<0.05) on univariate analysis are entered in the multivariate analysis model. In addition, PDA, SGA and gestational age at birth were known important cofactors for weight growth of preterm infants [26, 27]. We also included these three factors in the multivariate analysis model, even not statistically significant in univariate analysis.” (Page 12, Line 176-181)

DATA DESCRIPTION

4. **RE: The authors still need to provide more clarity in the manuscript regarding the distinction between individual infants who contributed to the study vs milk samples included the study.** **Demographic data for the infants themselves should be reported somewhere, even if just in a supplementary file, rather than just the sample-level data shown in Table 1. The figure they show in response to my first review comment (detailing** **the number of data points each infant contributed) should be included in the manuscript or at least in some form of supplementary material.**

Ans: We have clarified that some infants contributed more than 1 weekly sample if their hospital stay was longer (Page 12, Line 181-182). Demographic data for included 99 infants was displayed in S3 Table and was cited in the manuscript (Page 14, Line 195-196). The number of data points each infant contributed was also reported in S2 Figure (supporting information) and was cited in the manuscript (Page 8, Line 110).

ADDITIONAL COMMENTS BY SECTION:
OVERALL:

5. **RE: The language still suggests causal relationships rather than association in a few places, for example the title (I suggest "association of macronutrients in human milk with growth..." rather than "impact of macronutrients on growth") and Conclusion (I suggest "positive association with" rather than "effect on" body weight gain), and see line 199 in Results.**

Ans: We have corrected the words in title, results, and conclusion (Line 2, Line 214, Line 332).

6. **RE: The authors still use the unclear term "catch-up growth" in several places (eg line 241, line 260, line 319).**

Ans: We have deleted the term in line 256, line 274, line 332.

ABSTRACT:

7. **RE: In Methods, "full enteral feeding" suggests no additional nutritional support so authors can remove "without parenteral nutrition support" which is redundant and creates confusion. Add a description of what the outcome variable was and how it was measured.**

Ans: We have deleted the redundant words (Line 38) and added the description about outcome variable (Line 41-42).

8. **RE: What do the authors mean by "highly" associated (line 48)?**

Ans: We have deleted the word “highly” in line 50.

INTRODUCTION:

9. **RE: I disagree with the statement that breastmilk is protective against postnatal growth failure (line 64); rather, formula-fed infants often grow faster than milk-fed infants, but breastmilk is preferred despite that because of its other protective effects (eg on NEC, neurodevelopment, obesity prevention).**

Ans: Thank you for your comment. According to the reference 1 (Section on B. Breastfeeding and the use of human milk. Pediatrics. 2012;129(3):e827-41), lower rate of long-term growth failure is due to lower NEC incidence. We agree with you that most studies revealed that breast-fed infants are leaner than formula-fed infants. We have deleted this part to avoid confusion (Line 65).

METHODS:

10. **RE: Clarify what criteria were used to determine if milk samples were "insufficient" (line 87)?**

Ans: Each weekly average data contains 14 human milk samples as shown in Figure 2. We defined insufficient human milk sample as more than one missing sample within a week. We have added description about that (Line 88-89)

11. **RE: Line 97 remove "(96.5%)" which is redundant (and remove "up to" in line 96 if the number is known).**

Ans: We have removed these redundant words (Line 100).

12. **RE: Line 135 clarify if the intake includes protein intake from fortifier too.**

Ans: Yes, the energy and protein amount from fortifier were included in the calculation of total protein intake (g/kg/d) and protein-to-energy (PE) ratio (g/100 kcal). We have clarified it in Line 137-138.

13. **RE: Line 151, I appreciate the description of bottle feeding and ventilator use in the table footnotes, but it should be included here in the text in the description of covariates.**

Ans: We have added the definition of bottle feeding and ventilator use in section 2.4 Cofactors (Line 158-162) as follows, “The cofactor of bottle feeding means oral feeding without an orogastric tube applied on some days of the week when samples were taken. Ventilator support was defined as noninvasive positive pressure ventilators or nasal continuous positive airway pressure was used on some days of the week when the sample was taken.”

14. **RE: Line 158, I believe the authors mean "32 to <37" weeks**

Ans: Yes. We have corrected this mistake. (Line 166)

15. **RE: Line 164, clarify which variables are exposure vs outcome variables**

Ans: We have modified the sentence as follows: “The main exposure variables (protein concentration in human milk and total protein intake amount) and outcome variable (body weight gain velocity) were all normally distributed.” (Line 172-174)

RESULTS:

16. **RE: Line 197-8, clarify the meaning of the effect size, eg if this is describing the results of Table 4, "After adjustment, each 1 g/kg/day of protein content in milk was associated with 3.6 g/kg/day greater weight gain." Where did the number 5.70 come from? Same comment for lines 200-201 regarding protein intake. Results should only be described one time so if the authors discuss the results of Tables 4 & 5 later, it doesn't also need to be included here.**
    Ans: For effect size estimation, under alpha level of 0.05 and 90% power, the sample size of this study can detect the primary outcomes as minimal as 5.70 g/100ml (protein concentration) and 2.28 g/kg/day (daily protein intake). It is describing the result of Figure 3 (without adjustment with models). I’m sorry about unclear description. To avoid confusion, we have removed this part (Line 212-213, 216).
17. **RE: Line 198, the authors state only protein was associated with weight gain, but the table shows an association with energy too.**
    Ans: We have deleted the statement “There were no other significant associations of human milk components with growth” (Line 213-214).
18. **RE: Lines 212-214: This description of choosing GEE belongs in the Methods section.**
    Ans: We have deleted the description in results section (Line 227-229).

DISCUSSION:

19. **RE: Line 170, do the authors mean "achieved" rather than "required" here?**
    Ans: Yes. I have corrected the word (Line 284).

TABLES

20. **RE: Table 1: Is PMA normally distributed? If not, may be useful to show median/interquartile range instead. Also, clarify what weight z-score means; was it at the start of the week or end or an average?**

Ans: the Kolmogorov–Smirnov test was used, and PMA is not normally distributed. It was reported as median (interquartile range) in table 1. We also clarified that z-score means the weight status in the first day of week (Line 203-204).

21. **RE: Table 2: Title could be clarified to "Macronutrient concentration in human milk samples (N=245)". I think it would improve clarity to move the PMA at time of sample from Table 1 to Table 2, if the authors agree.**
    Ans: Thank you for your suggestions. We have revised the title of table 2 according to your suggestion (Line 208). However, the PMA means the infant age at the time of growth measurement rather than the lactation stage. The human milk may be collected earlier by mothers and stored in refrigerator at −20°C. It means that the infant may be fed with colostrum (stored in refrigerator) when he was 1 month old. Otherwise, the results of table 2 contains some data of donor milk, and the lactation stage (PMA) is uncertain. So we are sorry about that we couldn’t clarify the PMA (lactation stage) in table 2.

FIGURES

22. **RE: Figures 1 & 2. Good additions to the paper.**

Ans: Thank you for your comment.

23. **RE: Figure 3. Does this show adjusted or unadjusted values?**

Ans: Figure 3 showed unadjusted values. We have added this explanation in figure legend. (Page 18, Line 223)

Again, thank you for giving us the opportunity to strengthen our manuscript with your valuable comments and queries. We have worked hard to incorporate your feedback and hope that these revisions persuade you to accept our submission.

Sincerely,

Yi-Hsuan Lin, MD

Corresponding Author

Ming-Chih Lin, M.D. Ph.D.

Division of Neonatology & Pediatric Cardiology

Department of Pediatrics,

Taichung Veterans General Hospital,

Taichung city, Taiwan

TEL: 886-4-23592525-5901

FAX: 886-4-23741359
