# Supplementary material for: The association of macronutrients in human milk with the growth of preterm infants
Source: PLoS One. 2020 Mar 26;15(3):e0230800. doi: 10.1371/journal.pone.0230800 (PMC7098608; doi:10.1371/journal.pone.0230800)
Supplement: Attachment — Submitted filename: Response to Reviewers.docx [file pone.0230800.s006.docx]

Dear Dr. Young:

Thank you for inviting us to submit a revised draft of our manuscript entitled, “Association of macronutrients in human milk with the growth of preterm infants” to PLOS ONE again. We also appreciate the time and effort you and each of the reviewers have dedicated to providing insightful feedback on ways to strengthen our paper. Thus, it is with great pleasure that we resubmit our article for further consideration. We have incorporated changes that reflect the detailed suggestions you have graciously provided. We also hope that our edits and the responses we provide below satisfactorily address all the issues and concerns you and the reviewers have noted.

To facilitate your review of our revisions, the following is a point-by-point response to the questions and comments.

**To Reviewer #2:**

**Thank you for your comments and suggestions. The questions are answered below:**

1. **RE: A few minor points:
   Line 35: Change "enrolled" to "****is based on data from"
   Line 38: Change "from" to "of"
   Line 53: Change "have" to "had"
   Line 68: Change "higher" to "high"
   Line 69: Change "might not" to "are**
   Ans: Thank you for these comments. We have corrected these words.
2. **Line 157: Does "noninvasive" mean "non-intubated"?**
   Ans: Yes. Noninvasive positive pressure ventilators (NIPPV) mean non-intubated ventilator support from nasal prong. It also called nasal intermittent positive pressure ventilators (NIPPV). We have replaced the term with nasal intermittent positive pressure ventilators (Line 157).
3. **Line 140-142: Should read: "****Weight growth velocity was obtained by subtracting the first from the last weight of the week divided by 7"**

Ans: We are sorry about unclear description. We have revised this sentence (Line 141-142). The equation of weight growth velocity was displayed in Fig 2.

**To Reviewer #3:**

**Thank you for your comments and suggestions. The questions are answered below:**

1. **RE: Interquartile ranges should be presented as: median (25%ile, 75%ile) or the authors can present the range as median (min, max); but not usually median (#) as currently shown because it is unclear what the # represents**

Ans: We have revised it. The interquartile ranges are reported as median (25%ile, 75%ile) (Table 1).

2. **RE: Check the % of singletons in Supplementary Table 3; it doesn't match the N given**

Ans: We are sorry about this error. We have corrected it (Supplementary Table 3). 67.7% of enrolled infants were singletons.

3. **RE: There are a few very minor grammatical errors so the authors should proofread the manuscript for attention to English language.**

Ans: We have corrected some grammatical errors. The whole manuscript was edited for English language and writing (section Acknowledgement, Line 330-331)

4. **RE: The discussion is somewhat repetitive and could be edited for better organization and clarity, but it does not contain any errors and could be published as is.**

Ans: Thank you for your suggestion.

Again, thank you for giving us the opportunity to strengthen our manuscript with your valuable comments and queries. We have worked hard to incorporate your feedback and hope that these revisions persuade you to accept our submission.

Sincerely,

Yi-Hsuan Lin, MD

Corresponding Author

Ming-Chih Lin, M.D. Ph.D.

Division of Neonatology & Pediatric Cardiology

Department of Pediatrics,

Taichung Veterans General Hospital,

Taichung city, Taiwan

TEL: 886-4-23592525-5901

FAX: 886-4-23741359
